# Curcumin Mitigates Microplastic-Induced Damage in Livestock and Poultry: Mechanistic Insights and Strategies for Sustainable Farming

**DOI:** 10.3390/vetsci12111043

**Published:** 2025-11-01

**Authors:** Yicheng Shi, Zhiyu Su, Shiying Zhu, Xinrui Zhao, Jiatao Zhou, Panting Wang, Han Xia, Xishuai Tong, Fang Lv, Jianhong Gu

**Affiliations:** 1 College of Veterinary Medicine, Yangzhou University, Yangzhou 225009, China; 222002114@stu.yzu.edu.cn (Y.S.);; 2 Jiangsu Co-Innovation Center for Prevention and Control of Important Animal Infectious Diseases and Zoonoses, Yangzhou 225009, China; 3 Joint International Research Laboratory of Agriculture and Agri-Product Safety, The Ministry of Education of PR China, Yangzhou University, Yangzhou 225009, China; 4 Institute of Veterinary Immunology & Engineering, Jiangsu Academy of Agricultural Sciences, Nanjing 210014, China; 5 China-South Africa Joint Laboratory for Prevention and Control of Major Animal Diseases, Nanjing 210014, China

**Keywords:** curcumin, microplastic toxicity, oxidative stress, gut-liver axis, sustainable livestock, phytogenic additives, nanodelivery

## Abstract

**Simple Summary:**

Microplastics and nanoplastics (MNPs) are emerging contaminants in livestock and poultry farming, posing risks to animal health and food safety. This review explores the potential of curcumin, a natural compound derived from turmeric, to counteract the harmful effects of MNPs. We found that curcumin protects animals by reducing oxidative stress and inflammation in key organs such as the liver and gut. However, its effectiveness varies among species and is limited by poor absorption. Advanced delivery systems, such as nano-encapsulation, could enhance its efficacy. Although these findings are promising, further research is needed to develop safe and effective curcumin-based strategies suitable for real-world farming conditions.

**Abstract:**

The pervasive contamination of microplastics and nanoplastics (MNPs) in livestock and poultry production systems represent a critical threat to animal health, productivity, and food safety. This review systematically evaluates the potential of curcumin, a natural polyphenol from *Curcuma longa*, to mitigate MNP-induced toxicity, drawing on evidence from 25 preclinical studies (2014–September 2025). We highlight that curcumin exerts broad-spectrum, dose-dependent protection primarily through a dual mechanism: the preventive activation of the Nrf2/ARE antioxidant pathway and the therapeutic suppression of NF-κB-driven inflammation. These actions collectively ameliorate oxidative stress, restore metabolic homeostasis (e.g., via the gut–liver axis), and reverse histopathological damage across key organs, including the liver, kidneys, and reproductive tissues. A major translational insight is the significant species-specific variation in curcumin bioavailability, which is substantially higher in poultry than in ruminants, necessitating the development of tailored delivery systems such as nanoencapsulation. While the preclinical data are compelling, translating these findings into practice requires robust clinical trials to establish standardized, safe, and effective dosing regimens for food-producing animals. This review concludes that curcumin presents a promising, sustainable phytogenic strategy to enhance the resilience of livestock and poultry systems against MNP pollution, directly contributing to the One Health goals of safeguarding animal welfare, food security, and environmental sustainability.

## 1. Introduction

MNPs have become pervasive in terrestrial and aquatic environments, posing a growing threat to animal health via disruption of biochemical homeostasis [1]. These particles originate both from the fragmentation of larger plastics and from engineered nanoscale plastic materials, and they can infiltrate biological systems through ingestion, inhalation, or dermal absorption [2,3]. In livestock and poultry production systems, MNPs can accumulate via contaminated feed, water, bedding, and infrastructure, leading to chronic, low-dose exposures that may gradually impair physiological resilience [4,5]. Preclinical studies have increasingly linked MNP exposure with oxidative stress, inflammatory responses, disruption of gut barrier integrity, immunotoxicity, metabolic disturbances, and reproductive or developmental damage across critical organs such as the liver, kidney, intestine, and testes [6,7,8].

Curcumin (diferuloylmethane), the bioactive constituent derived from *Curcuma longa*, has attracted significant interest because of its antioxidant, anti-inflammatory, anti-apoptotic, and cytoprotective properties [9,10]. Extensive work in both animal and cell models shows that curcumin mitigates oxidative damage and suppresses pro-inflammatory pathways induced by environmental toxins, such as heavy metals, mycotoxins, and organic pollutants [11,12,13,14]. More recently, a handful of studies have explored curcumin’s ability to attenuate MNP-induced toxicity; results suggest that it may activate the Nrf2/ARE axis, inhibit NF-κB signaling, support mitochondrial integrity, and enhance cellular autophagy to counteract MNP-triggered oxidative injury and tissue damage [15].

Yet despite this promise, the current body of literature remains fragmented and inconsistent in both scope and rigor. Studies differ widely in the types and sizes of MNPs used (e.g., polyethylene, polystyrene), curcumin formulations and delivery strategies, dosing regimens, and species or cell models. Many reports omit essential methodological details or fail to assess and report risk of bias. Crucially, very few focus on livestock or poultry species, limiting translation of findings into agricultural practice. No systematic review to date has integrated existing evidence on curcumin’s protective effects against MNP toxicity specifically in a veterinary or farm animal context.

Hence, in this paper we undertake a systematic review of preclinical research examining curcumin’s mitigation of MNP-induced toxicity. We aim to capture the diversity of MNP types, experimental models, and curcumin dosing/formulation strategies; to synthesize efficacy findings along with mechanistic insights; to evaluate methodological quality and bias; and to identify gaps and priorities for future investigations that could support translation into livestock and poultry health management.

## 2. Methodology

We performed systematic searches in PubMed and Google Scholar (2014–September 2025). Search terms combined “curcumin” AND (“microplastic” OR “nanoplastic” OR specific polymer names). Two reviewers independently screened titles/abstracts and full texts; disagreements were resolved by consensus (Supplementary Figure S1). We extracted study characteristics (author, year, species, MNP type and dose, curcumin dose and route, outcomes, etc.) into a predefined Excel sheet (Supplementary Table S1). Risk of bias was assessed using SYRCLE’s tool for animal studies. Due to heterogeneity in species, exposures, and endpoints, we performed a narrative synthesis; when outcome data were sufficiently homogeneous, we considered quantitative pooling.

## 3. Result and Discussion

### 3.1. Sources and Composition of MNPs

To systematically assess MNP sources, plastics are categorized into primary particles (intentionally manufactured) and recycled particles (degraded from existing products) [16]. Generally speaking, plastic particles are divided into primary particles and recycled particles: primary particles are particles that are intentionally produced by the manufacturing industry for various purposes (particles used in the manufacture of plastic products, abrasive beads or personal health products). Recycled particles are particles (washing clothes, tire wear, etc.) produced when plastic products or waste are decomposed or worn into the environment [17]. Exposure of plastic contaminants to physical, mechanical, chemical and biological processes, such as fragmentation, weathering, hydrolysis, ultraviolet radiation and biodegradation, produces microplastics [18]. Microplastic was defined as synthetic polymeric compounds < 5 mm in diameter, which can be divided into microspheres, particles, fragments, films, fibers and irregular shapes according to their shapes [19]. Plastic residues persist in the environment, especially in marine and aquatic ecosystems [20]. It is estimated that more than 68 per cent of plastic residues in the ocean come from untreated or improperly recycled waste fragments [21]. What should not be underestimated is biodegradable plastics [22,23]. Due to the increasing use of biodegradable plastics and incomplete biodegradation, the presence of MNPs in the environment is increasing [24,25]. The ecotoxicological effects of MNPs on marine animals and plants, invertebrates and plants have been well documented [26,27]. Plastics are ubiquitous in the environment, including the atmosphere, soil and water, which may mean that microplastics can enter the food chain and pose a threat to human and animal health [28]. The presence of MNPs in human feces confirms the existence of MNPs in our diet [29]. The results of studies on plastic particles in farmland in Germany demonstrate the importance of soil cycling, as conventional treatments have greater MNP pollution than aquatic ecosystems [30].

The main routes of MNP entry into human and animal bodies are as follows: (1) inhalation of plastic particles from synthetic textiles and contaminated outdoor air; (2) ingestion of contaminated food and water; (3) skin contact [29]. The main way for humans and animals to be exposed to microplastics is through food intake [31]. Based on food consumption, the estimated annual intake of microplastics per capita is 39,000 to 52,000 [32]. In Europe, the annual per capita exposure to microplastics from eating meat animals is estimated to be 11,000 [33]. According to a report on plastic items in livestock and poultry farms, plastic is widely used in different elements of livestock and poultry farming, such as water pipes, sinks, pipes for storing or transporting feed, and plastic bottles for storing drugs [34]. A study from farms in Pakistan noted the presence of MNPs in the gizzard and crops of poultry and the dominance of fragmented MNPs. Four types of polymers have been detected based on the chemical characteristics of the particles: polyvinyl chloride (PVC), low-density polyethylene (LDPE), polystyrene (PS) and polypropylene homopolymer (PPH) [35]. At present, the microplastic pollutants detected in environmental pollutants include polyethylene (PE), polypropylene (PP), polyvinyl chloride (PVC), polyacrylonitrile (PAN), polycarbonate (PC), polyester phthalic acid (PEst), poly (terephthalic acid) (PET) and polystyrene (PS) [36,37,38].

Livestock and poultry are in contact with all aspects of the production of plastic products (Table 1): PE is widely used in the production of feed packaging films, plastic bags, plastic bottles and agricultural films, accounting for 85% of the total output of MNPs [39]. Oral, inhaled and skin exposure are the most common ways for humans to encounter PE, and PE-NPs have been detected in human feces [40]. The annual synthesis capacity of PET can reach 70 million tons, which is mainly used in automotive, packaging, electronic and textile components [41]. Because PET is a kind of polymer material with the ability to regenerate itself by transforming from polymeric state to original monomer state, it is widely found in drinking water and groundwater, air, soil and sediment [42]. Inhalation and skin are the main ways of human exposure to PET [31]. PC is the most widely used polymer in the production of food packaging, feeding bottles and other baby containers, electronics, supplies for the construction industry, automobiles, agricultural greenhouses and optical products [43]. However, over time, PC degrade and are released into the environment [44]. Bisphenol A (BPA) is a monomer substance of PC that can synthesize isoestrogens, which are present in air, dust, soil, water, sewage, wild animals, food and beverages [45]. It is of great concern because of the repeated exposure of humans to BPA [46,47,48]. PS is a thermoplastic colorless polymer commonly used in the production of experimental containers and food packaging [49]. Taking advantage of the light weight of PS foam, it is often used in building walls and roofs, as well as refrigerators and freezers, and can account for 10% of plastics in contaminated water and sediments [50]. PS, like other plastic particles, can enter the human body through respiratory, skin and oral routes [12]. PAN is a synthetic fiber and semi-crystalline polymer with anti-mold and anti-light properties, which is widely used in home textiles [51]. PAN fiber is severely limited due to its tendency to release toxic hydrogen cyanide during combustion. As a result, acrylonitrile (AN) polymers containing phosphorus groups have been produced, which play a role due to their excellent flame-retardant properties [51]. AN is widely used in synthetic elastomers, fibers, plastics, dyes, and pharmaceuticals [52]. Previous studies have reported the presence of AN in the workplace environment, vehicle tail gases, cigarette smoke, drinking water, and food [53]. Inhalation is the main route of human exposure to AN, but oral and skin contact routes should not be ignored [52]. MNPs can pose a threat to livestock and poultry breeding and meat food safety due to their physical, chemical and microbial properties [54]. Physical risk usually refers to the fact that, due to the extremely small size of MNPs, they can cross biological barriers such as skin, gut, blood–brain barrier, blood–testis barrier and even placental tissue and cause direct damage [55]. Chemical risk refers to the presence of potentially harmful persistent additives or contaminants in MNPs, while microbial risk is related to microorganisms adhering to the surface of MNPs [56].

**Table 1:** Microplastic exposure pathways in livestock and poultry production.

Exposure Pathways	MNP Type	Source	Key Findings
Feed Production	Polyethylene (PE)	Plastic mulch films and irrigation pipes	PE-MPs (0.8–45 μm) detected in agricultural soil-crop systems; 0.1–1% accumulation in maize roots [57]
Water Systems	Polyethylene (PE)	Plastic water pipes and storage tanks	PE particles dominate (85%) in agricultural water systems; 24,000 MPs/L from plastic bottles [58,59]
Housing Environment	Polypropylene (PP)	Plastic feeders and flooring	PP fragments (50–500 μm) prevalent in barn air and dust [60]
Reproductive System	Polytetrafluoroethylene (PTFE)	Non-stick feed equipment coatings	PTFE exposure linked to reduced sperm motility (64%) and testicular damage (57%) [61]
Manure Management	Polyvinyl chloride (PVC)	Plastic manure storage systems	PVC MPs (1–5 mm) detected in 92% of compost samples [62]
Veterinary Product	Polystyrene (PS)	Plastic syringes and packaging	PS MPs (100–300 nm) in 78% of injectable veterinary products [61]

### 3.2. The Organ-Specific Protective Effect of Curcumin on the Biological Toxicity of MNPs

The biological damage caused by microplastics to livestock and poultry has been widely reported. While direct MNP studies are limited, evidence from plastic-derived monomers like BPA and Di(2-ethylhexyl) phthalate (DEHP) provides mechanistic insights into curcumin’s potential to counteract organ damage caused by pollution from plastic sources. The protective effects of curcumin against MNP toxicity manifest across multiple organ systems, each with characteristic injury pathways and response mechanisms. Supplementary Table S1 provides the detailed experimental parameters (species, MNP type/size, curcumin dose, exposure duration, outcomes) underlying the summary below.

#### 3.2.1. Digestive System Toxicity

In gastrointestinal models, MNPs disturb epithelial integrity, downregulate tight junction proteins (e.g., Zonula Occludens-1 (ZO-1), occludin), promote epithelial oxidative stress, and trigger inflammatory cytokine release (Interleukin-6 (IL-6), Tumor Necrosis Factor-alpha (TNF-α)) [63,64]. Curcumin rescues barrier function by restoring tight junction expression, reducing Reactive Oxygen Species (ROS) accumulation, and suppressing NF-κB and Mitogen-Activated Protein Kinase (MAPK) signaling cascades [65,66]. In some studies, curcumin also modulates gut microbiota composition, thus reducing endotoxin translocation and alleviating downstream hepatic injury (Supplementary Table S1). The combined antioxidant and anti-inflammatory actions help maintain mucosal architecture and function [45,66,67,68].

#### 3.2.2. Neurotoxic Toxicity

Brain tissues exposed to MNPs often exhibit elevated oxidative stress, neuronal apoptosis, microglial activation, and pro-inflammatory cytokine production [69]. In such settings, curcumin can cross the blood–brain barrier, reduce ROS levels, inhibit microglial activation, downregulate NF-κB/NLR Family Pyrin Domain Containing 3 (NLRP3) pathways, and preserve neuronal cell density [69,70]. Some experimental results indicate that curcumin can restore caspase-3 levels and the Bcl-2-associated X protein/upregulating B-cell Lymphoma 2 (Bax/Bcl-2) ratio to normal, suppress the expression of Interleukin-1 beta (IL-1β) and TNF-α, and thereby effectively alleviate synaptic damage (Supplementary Table S1) [69,70,71].

#### 3.2.3. Curcumin Attenuates Reproductive Toxicity Induced by MNPs

Reproductive organs are particularly vulnerable to MNP impacts: testicular histology shows tubular degeneration, reduced sperm count/motility, hormonal imbalances (testosterone, Luteinizing Hormone (LH), Follicle-Stimulating Hormone (FSH)), and increased apoptotic markers (Bax, caspase-3) [72,73]. Curcumin treatment alleviates these effects by restoring antioxidant enzyme activity (Superoxide Dismutase (SOD), Glutathione (GSH)), suppressing inflammatory mediators, and modulating apoptotic regulation (Bcl-2, downregulating Bax) [72]. In studies included in Supplementary Table S1, animals receiving nano-curcumin often exhibit more pronounced amelioration in sperm parameters and hormonal indices than those given crude curcumin [73,74,75,76].

#### 3.2.4. Osteolysis Toxicity

Although fewer in number, studies examining skeletal toxicity show that MNPs may disrupt bone remodeling, increase osteoclast activity, and elevate oxidative stress in bone tissue [67]. Curcumin counters these effects by inhibiting Receptor Activator of Nuclear Factor Kappa-B Ligand (RANKL)/ROS signaling, suppressing NF-κB, and promoting osteoblast survival [77,78,79]. In these models, curcumin administration reduced bone resorption markers, preserved trabecular microarchitecture, and attenuated bone loss (Supplementary Table S1).

#### 3.2.5. Immunotoxicity

Exposure to MNPs often provokes innate immune activation, elevating TNF-α, IL-6, and IL-1β, and triggering oxidative stress in immune cells. Curcumin’s immunomodulatory efficacy is evidenced by downregulation of NF-κB and NLRP3 signaling, decreased pro-inflammatory cytokines, and improved antioxidant defense (e.g., GSH, SOD) in immune tissues or blood [80,81]. Some included studies show recovery of lymphocyte counts and improved phagocytic function (Supplementary Table S1).

#### 3.2.6. Tumor Metastasis

Though less frequently studied, MNPs have been implicated in enhancing tumor cell invasiveness and metastatic potential—likely through oxidative stress, DNA damage, and an inflammatory milieu [82,83]. In relevant models, curcumin suppresses MNP-enhanced metastatic markers (e.g., Matrix Metalloproteinase-2/9 (MMP-2/9), Vascular Endothelial Growth Factor (VEGF)), attenuates epithelial–mesenchymal transition (EMT) (epithelial–mesenchymal transition), and reduces cancer cell migration/invasion in vitro or metastasis in vivo. The dampening of NF-κB and MAPK pathways is commonly implicated (Supplementary Table S1) [82,83,84,85].

#### 3.2.7. Other Complications

Beyond the organs listed above, in cardiovascular studies, curcumin nano-micelles significantly attenuated BPA-induced subchronic cardiotoxicity in rats, ameliorating histopathological damage, electrocardiographic abnormalities, and oxidative stress markers, while modulating p38 Mitogen-Activated Protein Kinase/c-Jun N-terminal Kinase (p38/JNK) and Protein Kinase B (PKB)/Extracellular Signal-Regulated Kinase (AKT/ERK) apoptotic pathways [86,87]. In metabolic systems, evidence from studies on BPA suggests that curcumin may mitigate MNP-induced toxicity by insulin resistance in hepatic models (LO2 and Hepatoma G2 (HepG2) cells) by restoring glucose consumption, suppressing inflammatory cytokine release, and inhibiting JNK/p38/NF-κB signaling pathways [88,89]. In the endocrine system, curcumin can restore the normal levels of serum-free T4 and serum-free T3 by reducing the accumulation of malondialdehyde and the consumption of reduced glutathione. Thus, it can alleviate the damage caused by DEHP to the thyroid gland [90]. These findings collectively underscore curcumin’s capacity to mitigate multi-organ complications through pleiotropic mechanisms, even under MNP-related stress (Supplementary Table S1).

### 3.3. Nanodelivery Advances of Curcumin

Curcumin (Supplementary Figure S2) has emerged as a promising therapeutic agent due to its multifaceted biological properties, including antioxidant, anti-inflammatory, and anticancer effects. Curcumin’s therapeutic promise in mitigating microplastic toxicity is tempered by inherent limitations such as poor solubility, rapid metabolism, and low bioavailability—challenges exacerbated by interspecies pharmacokinetic variations (Table 2) [91]. For instance, poultry exhibits a 3.2-fold higher curcumin absorption (AUC 0–24: 112 ± 15 μg·h/mL) compared to ruminants (35 ± 8 μg·h/mL), necessitating species-tailored formulations to optimize efficacy while minimizing tissue residues. Meanwhile, the application of curcumin is restricted by the usage regulations concerning feed additives in various countries (Supplementary Table S2). To address these limitations, Jiang et al. have developed advanced nanoformulations that enhance curcumin’s stability and delivery efficiency [92]. For instance, cerium-based nanozymes combined with curcumin (CECH) demonstrated superior ROS-scavenging capabilities and cardioprotective effects in sepsis-induced cardiac injury by inhibiting ferroptosis and inflammation. Similarly, the encapsulation of curcumin within pH-responsive, enzyme-responsive, or mucus-penetrating nanocarriers (such as polysaccharide microgels, protein self-assemblies, and liposomes) has significantly enhanced both its intestinal absorption and systemic bioavailability [51,93]. These nanoformulations not only improve curcumin’s pharmacokinetic properties but also facilitate synergistic therapeutic outcomes through combination with other agents (e.g., siRNA or chemotherapeutic drugs), effectively addressing drug resistance in malignancies such as colorectal carcinoma [94,95]. Moreover, curcumin-loaded nanoplatforms have shown efficacy in diverse applications, including antimicrobial wound healing [96], anti-psoriasis treatments [97,98], and ocular disease management [99], by leveraging targeted delivery and controlled release mechanisms. Despite these advancements, challenges remain in scaling up production, ensuring long-term safety, and optimizing clinical translation. Ongoing research focuses on refining nanotechnology-based delivery systems—such as gold nanoparticles [100,101], phytosomes, and covalent organic frameworks [102]—to further unlock curcumin’s potential in precision medicine and inflammatory disease management.

Nevertheless, despite the promising enhancements that nanodelivery systems offer for curcumin, we must maintain a balanced perspective by acknowledging several potential limitations. The complexity and cost associated with manufacturing nanocarriers may pose barriers to adoption in large-scale livestock operations [103,104]. The regulatory pathway for approving nanocarrier formulations in food-producing animals remains underdeveloped; long-term safety profiles, residue behavior, and systemic exposure have not been comprehensively assessed [105]. Moreover, the intrinsic toxicity of certain nanoparticle systems raises concern: their minute dimensions and high surface reactivity can trigger oxidative stress, inflammation, or DNA damage in biological tissues [106,107]. Indeed, in some studies, curcumin-loaded nanoparticles exhibited dose-dependent cytotoxicity under high concentrations or unfavorable carrier designs [108]. Furthermore, many preclinical reports focus on relatively short treatment durations and local effects, often neglecting the risks of bioaccumulation, chronic exposure, or genotoxicity [109]. For these reasons, optimization of nanocarrier composition, rigorous safety testing (especially in relevant livestock species), and regulatory evaluation are essential before recommending widespread use in agricultural settings.

**Table 2:** Livestock-specific application of curcumin.

Species	Optimal Dose (mg/kg feed)	Bioavailability (%)	Key Target Tissue	Residual Clearance (days)	Reference
Broilers	300–350	22.4 ± 3.1	Liver > Gizzard	5.2 ± 0.8	Jagannathan et al. (2012) [110] Zhang et al. (2018) [111]
Swine	200–250	8.7 ± 1.5	Kidney > Adipose	9.1 ± 1.2	Ghosh et al. (2014) [112]
Dairy Cattle	150–200	3.1 ± 0.9	Rumen > Milk	14.3 ± 2.4	Moudgil et al. (2022) [113]

### 3.4. Evaluation of the Protective Potential of Curcumin for Exposure to Environmental Microplastics in Livestock and Poultry Production and Breeding

#### 3.4.1. Mechanistic Insights and Translational Cautions

Curcumin offers protection against plastic particle-induced injury through multiple interconnected pathways: enhancement of antioxidant defense (notably via Nrf2/ARE), inhibition of inflammatory cascades (such as NF-κB/NLRP3), modulation of gut–liver crosstalk, and adjustment of miRNA or epigenetic regulatory networks. In vitro and rodent studies report that curcumin upregulates detoxifying enzymes like HO-1 and NQO1, mitigates ROS and lipid peroxidation, and suppresses pro-inflammatory mediators such as TNF-α or IL-1β. A review of curcumin–MNP interaction literature found that “except for one study, curcumin restored all oxidative and histopathological damages induced by MNPs to normal” [114].

Translation to food-producing animals remains tentative. Differences in metabolic rate, signal responsiveness, and tissue microenvironment among species may alter efficacy. Experimental mechanistic protocols often use high doses or pure polymer systems, unlike mixed-plastic, low-dose environmental exposures. Some pathways may saturate or be regulated differently within intact organisms. Without in vivo validation in target animal species under realistic exposure conditions, mechanistic conclusions must remain provisional.

#### 3.4.2. Nano-Curcumin Formulations: Opportunities and Constraints

Nanoformulation strategies address curcumin’s inherently low bioavailability [115,116]. Encapsulation in nanoparticles—liposomes, polymeric matrices, phytosomes or mesoporous composites—improves absorption, stability, and targeting. In pig nutrition research, dietary curcumin nanospheres (CNs) significantly enhanced gut health metrics (e.g., villus height, tight junction protein expression) and suppressed intestinal inflammatory markers (e.g., TNF-α) [117].

Farm-scale feed inclusion of nano-curcumin faces several constraints. Nanoformulated curcumin may degrade or lose stability during pelleting, mixing and storage. The safety of the nanocarrier itself cannot be assumed; chronic exposure might precipitate unintended immune or oxidative responses. Residue kinetics—distribution, persistence and clearance—of both curcumin and its carrier require rigorous characterization to support food safety. Heterogeneous regulatory regimes worldwide further complicate approval, especially for nanomaterials in feed.

#### 3.4.3. Operational Considerations Under Field Conditions

Curcumin (particularly in nanoform) currently belongs in controlled experimental settings rather than widespread commercial use [118]. Trial programs must include dose optimization, challenge with mixed plastic exposures, evaluations of growth, tissue health biomarkers, and simultaneous residue sampling in edible tissues. Only after safety and efficacy are demonstrated under controlled and semi-commercial settings should pilot-scale trials proceed under veterinary oversight.

Real-world exposure complexities demand that protocols account for mixed polymer types, aging states of particles, co-contaminant interactions, dietary context, and host microbiome interplay. Success in ideal laboratory conditions may not replicate under farm variability unless study designs explicitly integrate these layers of complexity.

#### 3.4.4. Integrated Challenges and Future Trajectory

Even with encouraging preliminary data, significant barriers stand between mechanistic promise and field deployment. Uncertainties in dose translation across species hinder confident extrapolation. Interactions within mixed-contaminant conditions (e.g., MNPs plus leached additives) remain largely untested, and curcumin efficacy might differ under such circumstances. Nanoformulation safety, fate and residue behavior in edible tissues pose regulatory and consumer safety concerns. Economically viable, stable, process-compatible production of nano-curcumin is far from assured. Moreover, evolving regulations over feed additives and nanomaterials demand a clear, proactive approval strategy. Bridging these gaps requires concerted, interdisciplinary projects spanning molecular validation, pharmacokinetics, toxicology, residue studies, and scaled farm trials before curcumin can reliably transition into practical mitigation in livestock and poultry systems [119].

## 4. Conclusions

In preclinical models, curcumin has consistently shown efficacy in mitigating MNP-induced damage across multiple organ systems via mechanisms such as antioxidation, anti-inflammation, and barrier modulation. Nevertheless, the translational potential of these findings for livestock and poultry production remains nascent. Several pivotal issues require resolution, notably interspecies pharmacokinetic disparities, the multifaceted nature of composite MNP exposures, and the incomplete characterization of the safety and residue dynamics of both curcumin and its advanced delivery systems. Practical challenges in field deployment further complicate its immediate adoption. Thus, the application of curcumin, particularly in nanoformulations, should presently be restricted to investigative settings. Prioritizing future research on in vivo dosing, ADME and residue studies in food animals, long-term trials with mixed toxicants, nanocarrier toxicology, and integrated pilot farm studies is imperative. Success in these areas is paramount for establishing curcumin as a validated, compliant, and practicable intervention within sustainable animal agriculture.

## Data Availability

No new data were created or analyzed in this study. Data sharing is not applicable to this article.

## Supplementary Materials

The following supporting information can be downloaded at https://www.mdpi.com/article/10.3390/vetsci12111043/s1, Figure S1: PRISMA Flow Diagram for Study Selection; Figure S2: The molecular structure of curcumin; Table S1: Summary of Curcumin’s Protective Effects in Organ-Specific MNPs Toxicity Models; Table S2: A comparative table of curcumin feed additive regulations in key countries around the world.
