# Supplementary material for: Curcumin Mitigates Microplastic-Induced Damage in Livestock and Poultry: Mechanistic Insights and Strategies for Sustainable Farming
Source: Vet Sci. 2025 Nov 1;12(11):1043. doi: 10.3390/vetsci12111043 (PMC12656898; doi:10.3390/vetsci12111043)
Supplement: Supplementary file 1 [file vetsci-12-01043-s001.zip › vetsci-3902220-supplementary.pdf]

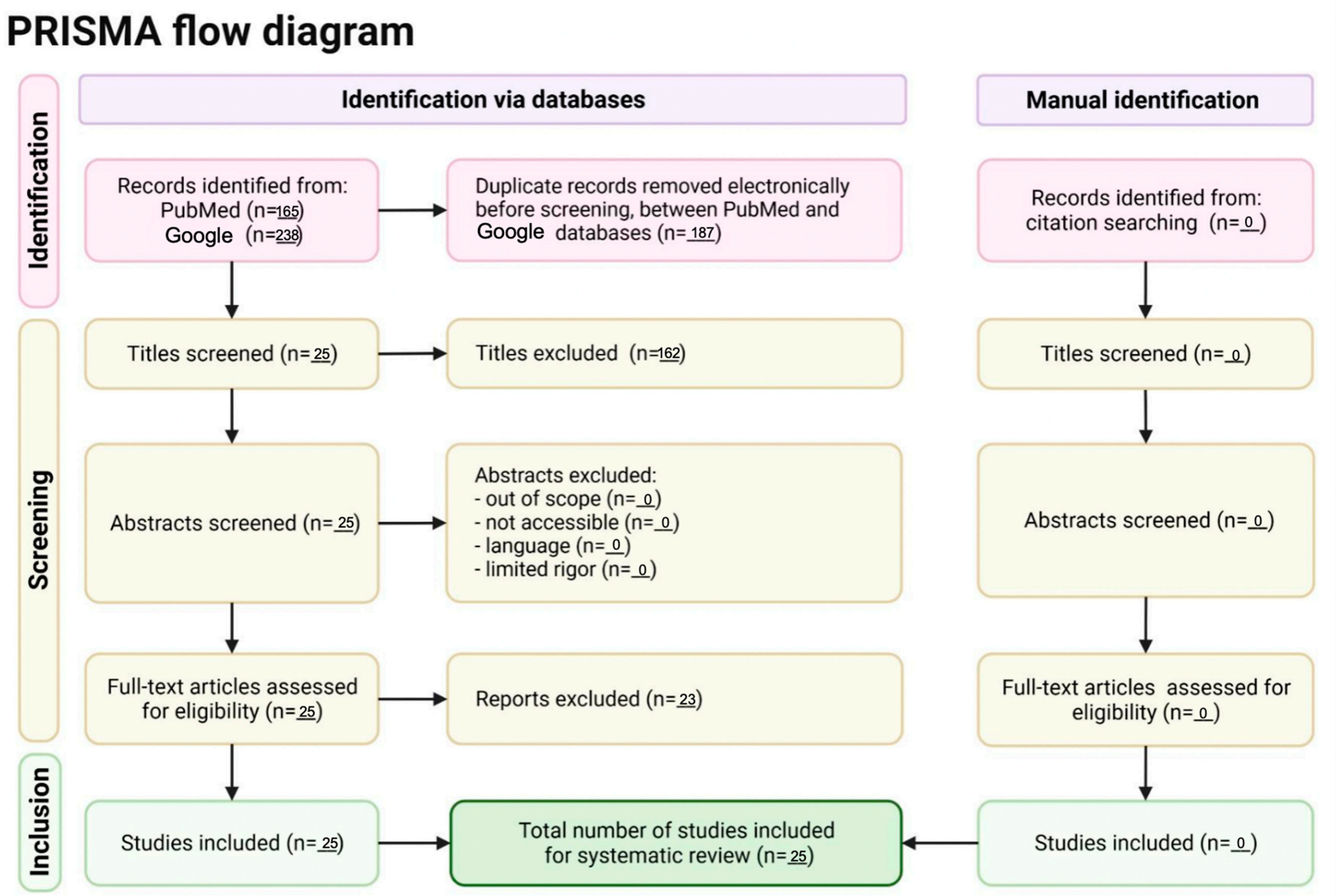


Supplementary Figure S1 PRISMA Flow Diagram for Study Selection

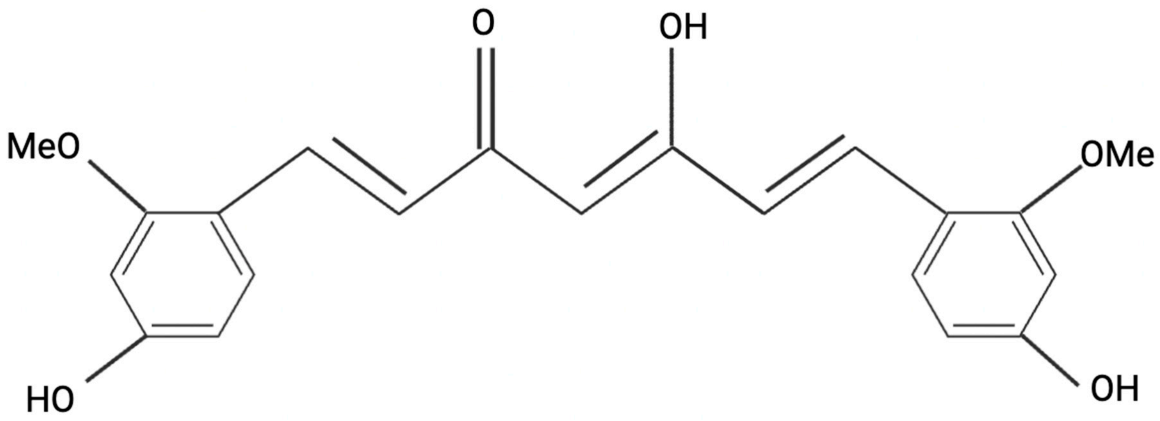


Supplementary Figure S2　The molecular structure of curcumin

**Supplementary Materials**

Supplementary Table S1 Summary of Curcumin's Protective Effects in Organ-Specific MNPs Toxicity Models

| Author (year) | Species / Model | MNPs Type & Size | Curcumin Formulation & Dose | Exposure Duration | Target Organ(s) | Key Measured Outcomes | Protective Effects of Observed |
|---|---|---|---|---|---|---|---|
| Ismail & El-Meligy (2022) [63] | 43 month albino rats | BPA | 200 mg/kg, p.o. | 30 days | stomach | PAS-positive reactivity, Bcl-2, PCNA | improvement in gastric structure |
| Hong et al. (2022) [66] | Male CD-1 mice | BPA | curcumin in the diet | 24 weeks | gut microbial and hepatic | ZO-1 and occludin, TLR4/NF-κB, serum lipopolysaccharide | reversed the down-regulation of ZO-1 and occludin, improved gut permeability, reduce d serum lipopolysaccharide, suppressed hepatic TLR4/NF-κB pathway activation |
| Amr et al. (2022) [64] | adult male albino rats | BPA | 100mg/kg, p.o. | 30 days | liver | TNF-α & IL-1,6,8 SOD, GPX and CAT levels, MDA and $H_2O2$ | exhibited anti-apoptotic, anti-inflammatory and potent antioxidant properties |
| Elswefy et al. (2020) [68] | Wistar male rats | BPA | 100 mg/kg, p.o. | 4 weeks | liver | IL-1β/IL-10, Bcl-2, caspase-3, MMP-9, MMP-2 | alleviated BPA-induced hepatic damage and fibrosis |
| Tiwari et al. (2016) [71] | Adult female Wistar rats | BPA | 20 mg/kg, i.p. | from PND7 to PND28 | hippocampus | LiCl and GSK-3β siRNA, Dkk-1, Wnt/β-catenin signaling pathway | provided neuroprotection against BPA-mediated impaired neurogenesis via activation of the Wnt/β-catenin signaling pathway |
| Abd El-Fattah et al. (2016) [73] | Male Wistar rats | DEHP | 200 mg/kg, p.o. | 30 days before and 45 days after DEHP administration | testis | TAC, GSH, MDA, ACP and ALP, LDH, Nrf2, HO-1, HSP60, HSP70 and HSP90, c-Kit protein | recovered injuries in biochemical, molecular and histological structures of testis; demonstrated intrinsic antioxidant properties and boosted Nrf2, HSP 60, HSP 70 and HSP 90 gene expression levels |
| Osanloo et al. (2017) [74] | Male NMRI mice | DEHP | 15 mg/kg, p.o. | 2 weeks | testis | AGD, free radicals | reduced pathological lesions in testicular tissue and maintained normal testosterone levels |
| Głombik et al. (2014) [72] | Male 12–36 weeks old mice | DEHP | 1 mM, 30 mM, 50 mM, 100 mM. p.o. | 21 days | testis | sperm motility, seminiferous tubule | exerted protective effects on sperm motility *in vitro* and mitigated seminiferous tubule injury *in vivo*. |
| Liang et al. (2021) [75] | Male Kunming mice | DBP | 100 mg/kg, p.o. | 30 days | kidney | oxidative stress, apoptosis, Bcl-2, Bax | reduced renal dysfunction, oxidative stress and apoptosis |
| Rihani L (2022) [76] | male rabbit | DBP | 200 mg/kg, p.o. | 30 days | testis | testosterone, count and motility of sperms, testis weight | recover DBP-induced injuries in spermatological parameters and histological structure of testis |

| | | | | | | | |
|---|---|---|---|---|---|---|---|
| An et al. (2018) [77] | RAW264.7 (monocyte/macrophage) cells; Male BALB/C mice | PE | 10, 20 μM (cells) ; 10 mg (mice) p.o. | 12 h (cells), 15 days (mice) | OC and bone | RANK/c-Fos/NFATc1 signaling pathway | prevented osteolysis and bone loss and inhibited the RANK/c-Fos/NFATc1 pathway |
| Cheng et al. (2017) [78] | Female 9-10 week old BALB/c mice | PMMA | 25 mg/kg, i.p. | 13 days | OC | TNF-α, IL-1β, IL-6, RANKL, osteoprotegerin messenger RNA | suppressed the RANKL signaling pathway |
| Liu et al. (2019) [79] | RAW264.7 macrophages; Male BALB/C mice | PE (40–48 μm) | 0, 1, 5, 10, 20 μM (cells); 10, 20 mg/kg (mice) | 2 days (cells), 14 days (mice) | OC and bone | ABCA1, ABCG1, and CAV1 | inhibited macrophage-mediated osteolysis and inflammation by promoting cholesterol efflux |
| Wang et al. (2011) [80] | HUVECs | DEHP | / | / | immune system | ICAM-1, IL-8, phosphorylation of ERK1/2 and p38 | inhibited ICAM-1 and IL-8 expression via ERK and p38 MAPK pathways |
| Wang et al. (2020) [81] | SPF male 5-week old BALB/c mice about 16.1 g | DBP | / | / | immune system | ROS, GSH, TAC, IL-1β, TNF-α, cytochrome C, caspase-8, caspase-9 and caspase-3 | Antagonized DBP-induced oxidative stress to reduce splenic injury |
| Tsai et al. (2020) [82] | carcinoma cells | DEHP | / | / | tumor | the aryl hydrocarbon receptor / ERK / SK1 / S1P3 signaling pathway | suppressed migration, invasion, and CSC-like cell maintenance through inhibition of the aryl hydrocarbon receptor/ERK/SK1/S1P3 signaling pathway |
| Li et al. (2014) [83] | MCF-7 human breast cancer cells | BPA | / | / | breast cancer | miR-19a and miR-19b, PTEN, p-AKT, p-MDM2, p53, proliferating cell nuclear antigen, | inhibited BPA-induced proliferation by modulating miR-19 / PTEN / AKT / p53 axis |
| Turgut et al. (2022) [84] | human colorectal adenocarcinoma cells | BPA | loaded polyvinylpyrrolidone K90 fibers, 17.37 mM | / | cancer cell | MDA. SOD-1, ER-α, ER-β | enhanced bioavailability, solubility, and anticancer efficacy |
| Heydari S et al. (2023) [85] | mouse RAW264.7 Cell | DEHP | 25 μM | / | cancer cell | MMPs 1, 8, 13 | inhibited MMPs gene expression, cancer cell metastasis, and establishment |
| Valokola et al. (2018) [86] | Rats | BPA | nanomicelle, 50 mg/kg | / | heart | phosphorylated p38, JNK, AKT, ERK1/2 | improved toxic effects of BPA |
| Apaydin et al. (2019) [87] | Adult male albino rats (250–300 g) | BPA | 100 mg/kg daily in olive oil | 28 days | heart | MDA, GPx、GST、SOD、CAT | minimized BPA-induced cardiotoxicity |
| Geng et al. (2018) [88] | human LO2 cells | BPA | 1 μM, 2.5 μM and 5 μM | 5 days | LO2 cells | MAPKs and NF-κB pathways, inflammatory cytokines and MDA | inhibited BPA-induced insulin resistance by suppressing the JNK pathway |
| Geng et al. (2017) [89] | human liver HepG2 cell | BPA | 1 μM, 2.5 μM and 5 μM | 5 days | liver HepG2 cells | JNK, p38, ERK, NF-κB pathways | inhibited JNK and p38 pathways, attenuating BPA-triggered insulin resistance |

| | | | | | | | |
|---|---|---|---|---|---|---|---|
| Sherif et al. (2021) [90] | male albino rats | DEHP | 15 mg/kg, p.o. | 30 days | thyroid | serum free T3, serum free T4, MDA, GSH | restore the normal levels of serum free T4 and serum free T3 by reducing the accumulation of malondialdehyde and the consumption of reduced glutathione |

**Note:** Acid Phosphatase (ACP), Alkaline Phosphatase (ALP), Anogenital Distance (AGD), ATP-binding cassette transporter A1 (ABCA1), ATP-binding cassette transporter G1 (ABCG1), B-cell Lymphoma 2 (Bcl-2), Bcl-2-associated X protein (Bax), Bisphenol A (BPA), Catalase (CAT), c-Jun N-terminal Kinase (JNK), Di(2-ethylhexyl) phthalate (DEHP), Caveolin-1 (CAV1), Dibutyl phthalate (DBP) , Dickkopf-related protein 1 (Dkk-1), Estrogen Receptor α,β (ER-α, β ), Extracellular Signal-Regulated Kinase 1/2 (ERK1/2), Glutathione (reduced) (GSH), Glutathione Peroxidase (GPX), Glutathione S-Transferase (GST), Glycogen Synthase Kinase-3 beta (GSK-3β), Heat Shock Protein 60, 70, 90 (HSP60, 70, 90), Heme Oxygenase-1 (HO-1), Hepatoma G2 (HepG2), Hydrogen Peroxide ($H_2O_2$), Polyethylene (PE), Human umbilical vein endothelial cells (HUVECs), Intercellular Adhesion Molecule-1 (ICAM-1), Interleukin-1, 6, 8, 1β, 10 (IL-1, 6, 8, 1β, 10), Lactate Dehydrogenase (LDH), Lithium Chloride (LiCl), Malondialdehyde (MDA), Mitogen-Activated Protein Kinase (MAPK), Matrix Metalloproteinase (MMP), Nuclear factor erythroid 2-related factor 2 (Nrf2), Nuclear Factor of Activated T-cells Cytoplasmic 1 (NFATc1), Nuclear Factor Kappa-B (NF-κB), Osteoclast (OC), p38 Mitogen-Activated Protein Kinase (p38), Periodic Acid-Schiff (PAS), Phosphatase and Tensin Homolog (PTEN), Phosphorylated AKT (p-AKT), Phosphorylated Mouse Double Minute 2 homolog (p-MDM2), Poly(methyl methacrylate) (PMMA), Proliferating Cell Nuclear Antigen (PCNA), Protein Kinase B (AKT), Receptor Activator of Nuclear Factor Kappa-B (RANK), Receptor Activator of Nuclear Factor Kappa-B Ligand (RANKL), Reactive Oxygen Species (ROS), Sphingosine Kinase 1 (SK1), Sphingosine-1-phosphate receptor 3 (S1P3), Superoxide Dismutase (SOD), Total Antioxidant Capacity (TAC), Toll-like Receptor 4 (TLR4), Tumor Necrosis Factor-alpha (TNF-α), Tumor protein p53 (p53), Wingless-related integration site (Wnt), Zonula Occludens-1 (ZO-1)

Supplementary Table S2 A comparative table of curcumin feed additive regulations in key countries around the world

| Country/Region | Regulatory Authority | Key Regulation | Classification System | Approval Process | Residue Limits (Example) | Penalties | Source |
|---|---|---|---|---|---|---|---|
| China | Ministry of Agriculture (MARA) | - Curcumin is regulated under feed additive standards (e.g., GB 2760-2014 for food additives, referenced for feed applications).<br>- Prohibited in fresh/unprocessed animal products unless explicitly approved. | Feed Additive (Coloring Agent) | Requires safety evaluation, registration, and compliance with national standards (e.g., GB 1886.76-2015). | No specific residue limits for feed, but food additive standards apply (e.g., ≤0.7 g/kg in certain foods). | Fines up to ¥50,000 and/or criminal charges for non-compliance with residue limits | CAAS |
| EU | European Food Safety Authority (EFSA) | - Approved as a food additive (E100) with ADI of 3 mg/kg body weight/day.<br>- Restricted in feed to avoid exceeding ADI through carry-over to animal products. | Food Additive (E100) | Requires EFSA safety assessment and authorization under Regulation (EC) No 1831/2003. | No specific feed limits, but residues in food must align with ADI | Market withdrawal, fines, or trade restrictions for non-compliance | EFSA Journal |
| USA | Food and Drug Administration(FDA) | - GRAS (Generally Recognized as Safe) status for specific uses (GRN No. 822, 686, 460).<br>- Permitted in feed as a coloring or flavoring agent under FDA CFR Title 21. | GRAS / Food Additive | Self-affirmed GRAS or FDA notification process; requires safety data submission. | No explicit limits, but must comply with Good Manufacturing Practices (GMP). | Product seizure, fines, or injunctions for unapproved use | FDA GRAS Notices |
| Japan | Ministry of Agriculture, Forestry and Fisheries(MAFF) | - Listed as an "Existing Food Additive" .<br>- Permitted in feed as a coloring agent but restricted in unprocessed animal products. | Existing Food Additive | Notification-based system under the Food Sanitation Act. | No explicit residue limits; adherence to general food safety standards required. | Fines up to ¥1,000,000 and product recalls | MAFF Guidelines |
| South Korea | Ministry of Food and Drug Safety(MFDS) | - Classified as a food additive (coloring agent).<br>- Prohibited in feed for unprocessed meat, fish, or poultry. | Food Additive (Coloring Agent) | Requires pre-market approval and compliance with Korean Food Standards Codex. | Maximum 60 mg/serving in processed foods; no specific feed limits | Fines up to ₩50 million and product bans | MFDS |
